# Linezolid Population Pharmacokinetic Model in Plasma and Cerebrospinal Fluid Among Patients With Tuberculosis Meningitis

**DOI:** 10.1093/infdis/jiad413

**Published:** 2023-09-22

**Authors:** Noha Abdelgawad, Sean Wasserman, Mahmoud Tareq Abdelwahab, Angharad Davis, Cari Stek, Lubbe Wiesner, John Black, Graeme Meintjes, Robert J Wilkinson, Paolo Denti

**Affiliations:** Division of Clinical Pharmacology, Department of Medicine, University of Cape Town, South Africa; Centre for Infectious Diseases Research in Africa, Institute of Infectious Disease and Molecular Medicine, University of Cape Town, South Africa; Institute for Infection and Immunity, St George's University of London, United Kingdom; Division of Clinical Pharmacology, Department of Medicine, University of Cape Town, South Africa; Centre for Infectious Diseases Research in Africa, Institute of Infectious Disease and Molecular Medicine, University of Cape Town, South Africa; The Francis Crick Institute, London, United Kingdom; Faculty of Life Sciences, University College London, United Kingdom; Centre for Infectious Diseases Research in Africa, Institute of Infectious Disease and Molecular Medicine, University of Cape Town, South Africa; Division of Clinical Pharmacology, Department of Medicine, University of Cape Town, South Africa; Department of Medicine, Walter Sisulu University, Mthatha, South Africa; Centre for Infectious Diseases Research in Africa, Institute of Infectious Disease and Molecular Medicine, University of Cape Town, South Africa; Department of Medicine, University of Cape Town, South Africa; Centre for Infectious Diseases Research in Africa, Institute of Infectious Disease and Molecular Medicine, University of Cape Town, South Africa; Division of Infectious Diseases and HIV Medicine, Department of Medicine, University of Cape Town, South Africa; The Francis Crick Institute, London, United Kingdom; Department of Infectious Diseases, Imperial College London, United Kingdom; Division of Clinical Pharmacology, Department of Medicine, University of Cape Town, South Africa

**Keywords:** cerebrospinal fluid, linezolid, modeling and simulation, population pharmacokinetics, tuberculosis meningitis

## Abstract

**Background:**

Linezolid is evaluated in novel treatment regimens for tuberculous meningitis (TBM). Linezolid pharmacokinetics have not been characterized in this population, particularly in cerebrospinal fluid (CSF), as well as, following its co-administration with high-dose rifampicin. We aimed to characterize linezolid plasma and CSF pharmacokinetics in adults with TBM.

**Methods:**

In the LASER-TBM pharmacokinetic substudy, the intervention groups received high-dose rifampicin (35 mg/kg) plus 1200 mg/day of linezolid for 28 days, which was then reduced to 600 mg/day. Plasma sampling was done on day 3 (intensive) and day 28 (sparse). A lumbar CSF sample was obtained on both visits.

**Results:**

Thirty participants contributed 247 plasma and 28 CSF observations. Their median age and weight were 40 years (range, 27–56) and 58 kg (range, 30–96). Plasma pharmacokinetics was described by a 1-compartment model with first-order absorption and saturable elimination. Maximal clearance was 7.25 L/h, and the Michaelis-Menten constant was 27.2 mg/L. Rifampicin cotreatment duration did not affect linezolid pharmacokinetics. CSF-plasma partitioning correlated with CSF total protein up to 1.2 g/L, where the partition coefficient reached a maximal value of 37%. The plasma-CSF equilibration half-life was ∼3.5 hours.

**Conclusions:**

Linezolid was readily detected in CSF despite high-dose rifampicin coadministration. These findings support continued clinical evaluation of linezolid plus high-dose rifampicin for the treatment of TBM in adults.

***Clinical Trials Registration.*** ClinicalTrials.gov (NCT03927313).

## Teaser

We describe linezolid plasma and cerebrospinal fluid pharmacokinetics in adults with tuberculosis meningitis/HIV. Linezolid was readily detected in cerebrospinal fluid despite coadministration with high-dose rifampicin. Our findings support continued clinical evaluation of linezolid plus high-dose rifampicin for the treatment of tuberculosis meningitis in adults.

Tuberculous meningitis (TBM) is the most fatal and debilitating form of tuberculosis, with a particularly high burden among people living with HIV [1]. One reason for severe outcomes is that the current regimen for TBM is based on treatment for pulmonary tuberculous (TB) and may result in suboptimal central nervous system (CNS) concentrations [2]. Drugs targeted at TBM should cross several barriers to reach the site of disease, including the blood-brain barrier and the blood–cerebrospinal fluid (CSF) barrier, which separate systemic circulation from their site of action in the CNS. These barriers pose a therapeutic challenge by limiting entry of drugs into the CNS. Moreover, disease-related changes in blood-brain barrier permeability and dynamic changes in protein concentrations may have important implications for drug penetration into the brain [3].

Linezolid, an oxazolidinone antibiotic, is highly effective for the treatment of drug-resistant pulmonary TB. Linezolid is also used to treat gram-positive bacterial infections in the CNS [4–6], where good drug penetration has been documented, making it an attractive candidate for TBM treatment [7–9]. Small observational studies have shown improved clinical parameters with linezolid use in children and adults with TBM [10, 11]. Based on these encouraging observations, linezolid is being investigated as part of intensified antibiotic therapy in several clinical trials for TBM [12].

Specific features of TBM may influence the pharmacokinetics (PK) of linezolid, with potential implications for safety and efficacy, given its narrow therapeutic window. These include host factors (eg, body size) and disease factors, such as CSF protein concentrations and blood-brain barrier permeability. Also, clinical trials pair linezolid with high-dose rifampicin in TBM treatment regimens. As a potent inducer of the cytochrome P450 (CYP) system and upregulator of drug transporters [13], rifampicin could affect the PK of linezolid. Studies in healthy volunteers and pulmonary TB have shown a moderate reduction in linezolid exposure when administered with standard-dose rifampicin [14, 15]. The impact on site of disease (CSF) concentrations and clinical implications of this PK interaction is unknown but could theoretically lead to suboptimal treatment or the development of antimicrobial resistance.

The objectives of this analysis were to describe the PK of linezolid in the plasma and CSF of adults with TBM to explore the effect of high-dose rifampicin on linezolid PK, evaluate covariate effects on plasma and CSF drug levels, and simulate exposures for optimized dosing strategies.

## METHODS

### Study Data

This was a substudy of LASER-TBM [16], a phase IIb open-label trial that evaluated the safety and PK of intensified antibiotic therapy in adults with HIV and TBM [12]. Participants were enrolled from 4 public hospitals in Cape Town and Gqeberha, South Africa, and randomized to study interventions within 5 days of starting antituberculosis treatment. The standard-of-care group (control) received fixed-dose combination oral tablets (rifampicin, 10 mg/kg; isoniazid, 5 mg/kg; pyrazinamide, 25 mg/kg; ethambutol, 15 mg/kg) according to World Health Organization weight bands. Participants allocated to experimental groups were administered the standard regimen with a higher dose of rifampicin (35 mg/kg in total with bespoke weight bands [17]) and linezolid for 56 days (1200 mg once daily for the first 28 days, then 600 mg once daily), with or without aspirin. All participants received adjunctive dexamethasone.

PK sampling visits were scheduled on day 3 (±2 days) and day 28 (±2 days) after study entry. At the day 3 visit, plasma was collected at predose and 0.5, 1, 2, 3, 6, 8 to 10, and 24 hours postdose (intensive) and, on day 28, at predose and 2 and 4 hours postdose (sparse). Sparse sampling was performed on day 3 for participants who declined intensive sampling or for whom intensive sampling could not be done. One lumbar CSF sample was collected at each PK sampling visit, with timing randomized to intervals of 1 to 3, 3 to 6, 6 to 10, and 24 hours after dosing. Clinical information was collected, and full blood count and serum chemistry data were obtained at each visit. Total protein, albumin, and glucose were measured in CSF samples.

Linezolid plasma and CSF concentrations were measured in the Division of Clinical Pharmacology at the University of Cape Town. The plasma assay summary has been described [18]. Cholesterol and 4-β hydroxy cholesterol (4β-OHC) were also measured in predose plasma samples collected on both PK visits. 4β-OHC is a metabolite of cholesterol formed by CYP3A4, and the ratio between its concentration and that of cholesterol is used as a marker of CYP3A4/5 endogenous activity [19]. Additionally, the unbound concentration of linezolid in plasma was quantified in a subset of samples to estimate the degree of plasma protein binding. Details of analytic assays are outlined in the supplementary file.

Informed consent was obtained from all participants or their proxies. The study was approved by the University of Cape Town Human Research Ethics Committee (reference 293/2018), Walter Sisulu University Human Research Ethics Committee (reference 012/2019), and the South African Health Products Regulatory Authority (reference 20180622). The trial is registered on ClinicalTrials.gov (NCT03927313).

### PK Modeling

Nonlinear mixed-effects modeling was used to create a population PK model describing linezolid PK in plasma and lumbar CSF. The model was developed sequentially: first describing plasma linezolid and then including CSF concentrations.

For the plasma PK, we tested 1- and 2-compartment disposition models with linear or saturable elimination and first-pass effect. The CSF concentrations were described via a hypothetical effect compartment linked to the central (plasma) compartment, which estimates the first-order equilibration rate constant of linezolid between the central and effect compartments (*k*_plasma-CSF_) and the pseudo-partition coefficient (PPC). Further details on the modeling approach are available in the supplementary file.

Following the development of the structural model, we tested the effect of potential covariates: creatinine clearance (calculated with the Cockcroft-Gault equation [20]), age, study visit, duration of concomitant rifampicin treatment, study site, and treatment arm. For the CSF PK parameters PPC and *k*_plasma-CSF_, we also tested the effect of CSF total protein, albumin, and glucose concentrations. The precision of the parameter estimates of the final model, expressed as 95% CIs and percentage relative SE, was assessed via sampling/importance resampling [21].

In the plasma samples with matched free and total linezolid concentrations available, the free concentrations were regressed against the total concentration with an intercept of 0 per Deming regression [22, 23]. The fraction unbound (fu) was estimated from the slope of the regression line.

### Simulations

The model-derived area under the concentration-time curve from time 0 to 24 hours postdose (AUC_0–24h_) and the concentration at 24 hours postdose (C_24h_) were calculated for the available profiles. Monte Carlo simulations (n = 10 000) were performed with final model parameters to simulate concentration-time profiles in plasma and CSF following daily linezolid doses of 600 or 1200 mg at steady state for a typical participant with a median fat-free mass of 45 kg and CSF protein of 0.995 mg/mL.

## RESULTS

### Study Data

Thirty participants underwent PK sampling on day 3 of the study (the first PK visit), and 18 had PK sampling on day 28 (the second PK visit)—1 of whom was excluded from this analysis because all 3 samples were below the limit of quantification (BLQ; later confirmed to have missed dosing). Reasons for missing the second PK visit included death, interrupting linezolid dose due to adverse events, or withdrawing consent. Concentrations available for PK modeling totaled 247 for plasma (6 BLQ, 2.43%) and 28 for CSF (7 BLQ, 25%). All participants were receiving 1200 mg of linezolid daily at the first PK visit; on day 28, 13 received 1200 mg and 4 received 600 mg. Baseline clinical characteristics are summarized in Table 1.

**Table 1.** Clinical Characteristics by Visit Day

	Median (Range) or No. (%)	
	Day 3 (n = 30)	Day 28 (n = 17)
Sex: male	18 (60)	11 (65)
Age, y	40 (27–56)	37 (27–51)
Weight, kg	58 (30–96)	61 (37–81)
Height,^a,b^ m	1.61 (1.48–1.80)	1.61 (1.57–1.80)
Fat-free mass,^b,c^ kg	45 (30–59)	48 (32–60)
Serum creatinine, mmol/L	61 (27–87)	50 (34–86)
4β-OHC/cholesterol,^d^ molar ratio × 10^−5^	1.48 (0.313–6.79)	1.90 (0.384–5.50)
Daily linezolid oral dose, mg		
1200	30	10
600	0	7
Duration of rifampicin treatment,^e^ d	5 (0–7)	30 (27–38)
CSF^f^		
Total protein, g/L	1.46 (0.310–54.7)	0.750 (0.220–2.19)
Albumin, g/L	3.32 (0.93–23.34)	4.47 (0.46–11.41)
Glucose, mmol/L	2.9 (1.0–5.3)	3.2 (2.2–3.6)
Antiretroviral therapy		
Previous	11 (37)	6 (35)
Naive	10 (33)	5 (29)
Undergoing	9 (30)	6 (35)
Participants concomitantly taking		
Tenofovir/emtricitabine/efavirenz	7	5
Abacavir/lamivudine/lopinavir	2	1

Abbreviations: 4β-OHC, 4-β hydroxy cholesterol; CSF, cerebrospinal fluid.

^a^Heights were missing for 18 (60%) of 30 participants and imputed by sex and weight as outlined in the supplementary file.

^b^Data represent the nonmissing values (ie, data do not include the imputed values).

^c^Fat-free mass was calculated by sex, weight, and height according to the formula of Janmahasatian et al [24].

^d^The ratio of 4β-OHC to cholesterol was missing in 4 and 3 participants on days 3 and 28, respectively.

^e^The total number of days since the start of tuberculosis treatment, which was ∼1 to 3 days before recruitment into the study and start of the investigational treatment. When starting treatment, participants took the standard dose of rifampicin (10 mg/kg) and then switched to a high dose (35 mg/kg) at the start of the study.

^f^Participants with CSF observations, CSF protein, albumin, and glucose were missing for 2/18 (11%) on day 3 and for 3/10 (30%) on day 28.

Median CD4 count was 137 cells/mm^3^ (range, 2–890). Median duration on rifampicin therapy was 5 days (range, 0–7) at the day 3 PK visit and 30 days (range, 27–38) at the day 28 visit. Median CSF total protein concentrations decreased from 1.46 g/L (range, 0.31–54.7) at day 3 to 0.75 g/L (range, 0.22–2.19) at day 28.

### PK Modeling

**Figure 1.**
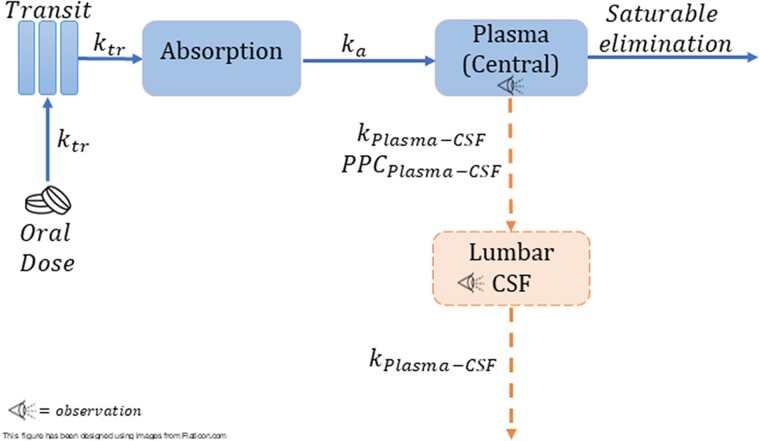
Representation of the final model: *k*_tr_ is the rate constant for the drug passage through the transit compartments; *k*_plasma-CSF_ is the equilibration rate constant for plasma–cerebrospinal fluid (CSF), which describes how soon the change in plasma is reflected in the CSF; *k*_a_, absorption rate constant; PPC_plasma-CSF_ is the pseudo-partition coefficient, which represents the ratio of drug in CSF to the plasma.

The plasma PK of linezolid was best characterized by a 1-compartment disposition model, saturable elimination with Michaelis-Menten, and first-order absorption preceded by a chain of transit compartments. Saturable elimination resulted in a better model fit than linear elimination (drop in objective function value [dOFV] = −9.03, *P* = .00205, *df* = 1). A schematic diagram of the model in shown in Figure 1. Two-compartment disposition was tested but did not result in a significant improvement of fit. Maximal clearance (CLmax) and volume of distribution (V) were allometrically scaled with fat-free mass (dOFV = −30 vs −7.7 with total body weight). In a typical participant (median fat-free mass, 45 kg), the value of CLmax was 7.25 L/h; the Michaelis-Menten constant (*k*_m_), which is a parameter that governs saturable hepatic elimination and represents the linezolid concentration at which half the CLmax is reached, was 27.2 mg/L; and the V was 40.8 L. The inclusion of between-visit variability in CLmax improved the model fit, but no systematic increase or decrease was observed with duration of treatment. Longitudinal changes in clearance were explored by testing autoinhibition and duration of rifampicin cotreatment, but no significant effect was found for either. We also could not find any effect when testing the ratio of 4β-OHC to cholesterol, creatinine clearance, or age on CLmax and bioavailability (F). The final parameter estimates are presented in Table 2. A visual predictive check showing adequate model fit is depicted in Supplementary Figure 1.

**Table 2.** Final Population Pharmacokinetic Parameter Estimates for Linezolid in Plasma and Lumbar CSF

Parameter	Estimate (95% CI) [RSE, %]^a^
CLmax: maximal clearance, L/h^b^	7.25 (6.09–8.86) [9.93]
*k*_m_: Michaelis-Menten constant, mg/L	27.2 (16.0–46.4) [29.1]
V: volume of distribution, L^b^	40.8 (37.9–43.6) [3.65]
F: bioavailability	1 fixed
MTT: mean transit time, h	0.211 (.112–.342) [28.6]
NN: No. of absorption transit compartments	5.68 (2.36–11.8) [43.5]
*k*_a_: absorption rate constant, h^−1^	1.21 (.831–1.76) [19.6]
Plasma	
Proportional error, %	21.5 (18.8–24.7) [7.06]
Additive error, mg/L^c^	0.173 (.0379–.355) [47.1]
BSV: between-subject variability in CLmax, %	9.60 (3.44–13.9) [51.9]
BVV: between-visit variability in CLmax, %	20.3 (15.3–26.9) [30.7]
BOV: between-occasion variability in *k*_a_, %	87.9 (66.4–110) [25.9]
BOV in MTT, %	110 (75.8–144) [32.8]
*k*_plasma-CSF_: equilibration rate constant to CSF, h^−1d^	0.198 (.0849–.340) [33.7]
PPC_max_: maximal pseudo-partition coefficient to CSF	0.365 (.238–.566) [23.2]
CSF protein_max_: CSF protein at which PPC_max_ is reached, mg/mL^e^	1.18 (.730–1.90) [24.4]
CSF	
Proportional error, %	91.5 (63.3–151) [23.4]
Additive error, mg/L^c^	0.02 fixed

Abbreviations: CSF, cerebrospinal fluid; RSE, relative SE.

^a^95% CIs and RSEs were computed with sampling/importance resampling on the final model.

^b^CLmax and V were allometrically scaled, so the values reported here refer to the typical participant (ie, a median fat-free mass of 45 kg).

^c^The estimated additive component of the error was not significantly different from its lower boundary of 20% of the lower limit of quantitation, so it was fixed to this value.

^d^Corresponds to an equilibration half-life of 3.5 hours (95% CI, 2.04–8.16 hours).

^e^For CSF protein < CSF protein_max_ (ie, the breakpoint): PPC*_i_* = PPC_max_ · [slope · (CSF protein – breakpoint)], where the breakpoint was an estimated 1.18 mg/mL and the slope was 0.847. The slope was calculated from the following equation: slope = (amplitude – intercept) / (breakpoint – 0), where the intercept and amplitude were fixed to 0 and 1, respectively. For CSF protein ≥ CSF protein_max_: PPC*_i_* = PPC_max_. The PPC-CSF protein relationship is depicted in Figure 2, and more details are provided in the supplementary file.

**Figure 2.**
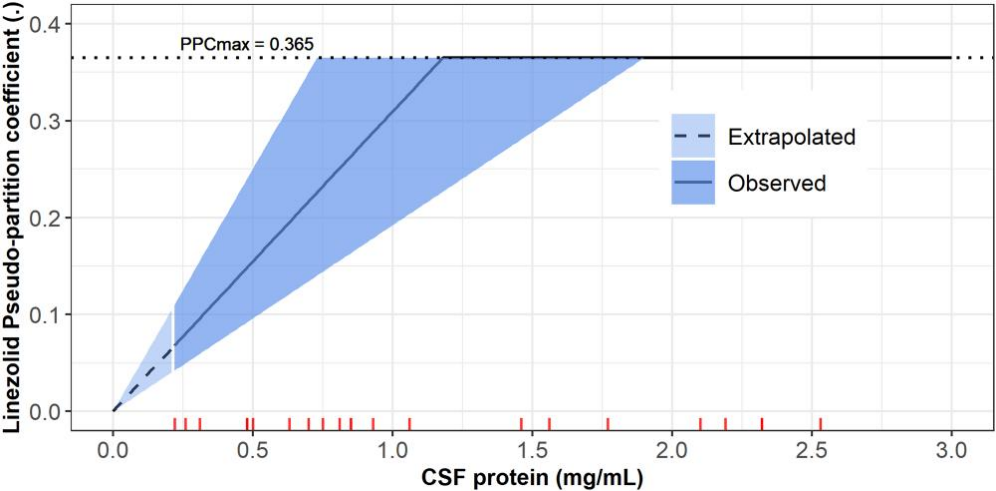
The relationship of the pseudo-partition coefficient (PPC) vs the cerebrospinal fluid (CSF) protein level via the piecewise function (broken stick). The solid line represents the median, and the shaded areas represent the uncertainty around the estimates of the breakpoint (the maximal CSF protein value at which PPC_max_ is reached) and the calculated slope. The dashed line depicts the extrapolated part of the PPC–CSF protein relationship for CSF protein values outside the range observed in the study cohort; the lowest observed value was 0.22 mg/mL. The ticks on the x-axis represent the values of the CSF protein observed in our cohort; CSF protein values >3 mg/mL were truncated for better figure visibility. Some ticks are overlapping because there are some duplicated CSF protein values.

The CSF concentrations were linked to the plasma concentrations with an equilibration half-life of 3.5 hours (95% CI, 2.04–8.16) and the steady-state equilibrium ratio (PPC), indicating the relative amount of linezolid exposure in CSF, which was dependent on CSF protein levels. Figure S2 shows the interpretability of the equilibration rate constant and the PPC in the context of effect compartment modelling approach. The PPC-CSF protein relationship was described by a piecewise linear function (broken stick), where the PPC increased with higher CSF protein levels until reaching a maximal CSF protein value where it plateaued (ie, a maximal PPC value). The breakpoint was estimated, while the slope (ie, the change in PPC per change in CSF protein) was calculated from the breakpoint and the intercept (minimum PPC), which was fixed at 0 to prevent the estimation of negative PPC values, which are physiologically unplausible. For each 0.1-mg/mL increase in CSF protein, we found an increase of 3% in PPC up to 1.18 mg/mL of CSF protein, after which the PPC reached a maximal value of 0.365 (95% CI, .238–.566; Figure 2**)**. CSF protein and albumin correlated significantly with PPC; note, however, that both are highly positively correlated. Only CSF protein was included in the final model because it resulted in a more significant dOFV and because albumin is a component of the proteins measured.

The regression plots of linezolid-free vs total concentrations and linezolid fu vs total linezolid concentration (milligram/liter) are shown in Supplementary Figures 3 and 4. There was no apparent trend of changing fu across the observed range of total linezolid concentration.

### Simulations

**Figure 3.**
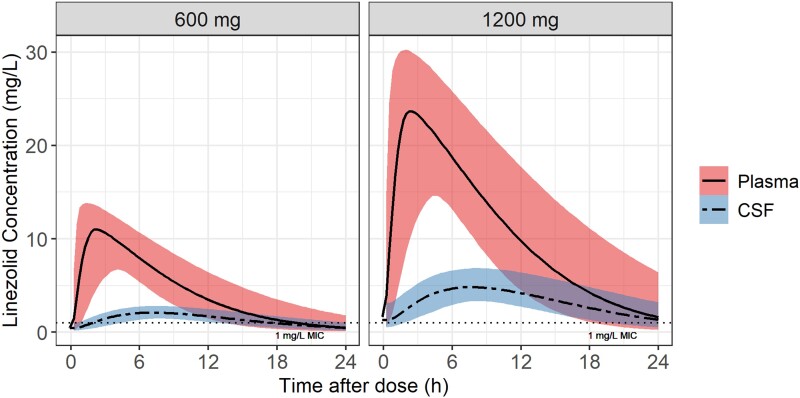
Simulated typical concentration-time profiles for plasma and cerebrospinal fluid (CSF) for the oral daily dose of linezolid: 1200 and 600 mg. The solid and dashed lines represent the median for the plasma and CSF, respectively, and the shaded areas represent the 90% CIs. The horizontal dotted line indicates the wild type minimum inhibitory concentration (MIC) value of linezolid for *Mycobacterium tuberculosis*.

**Figure 4.**
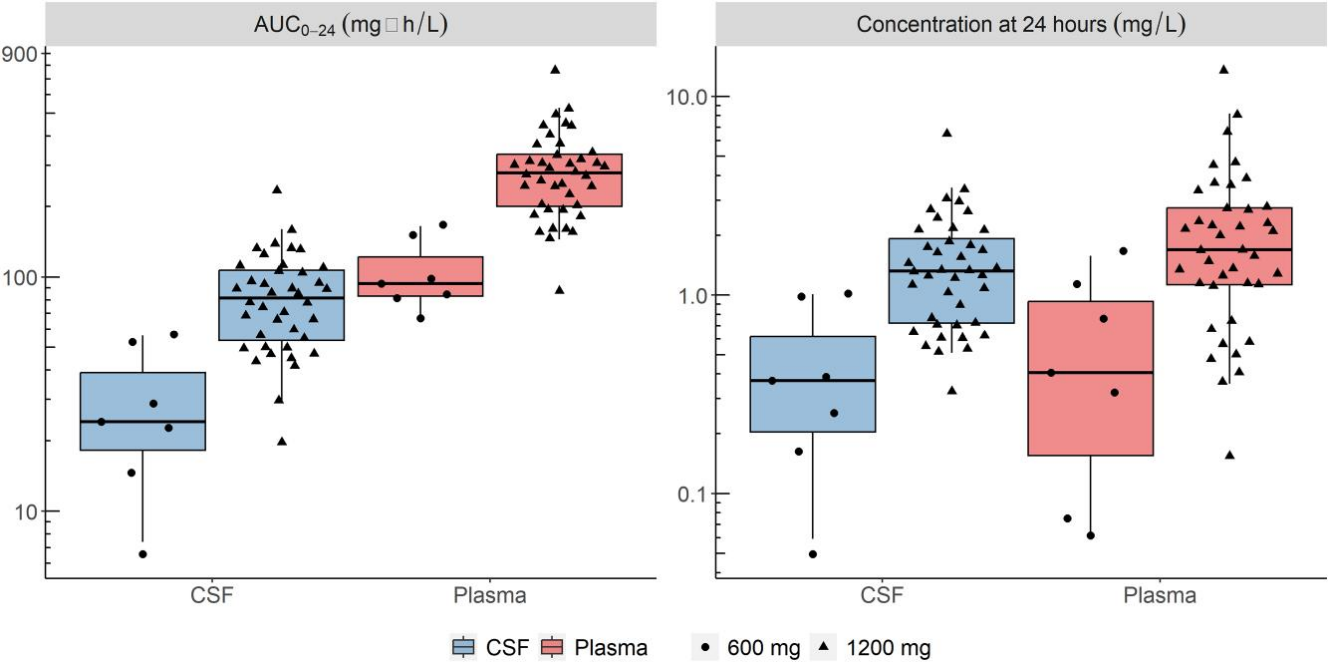
Secondary model-derived exposure parameters: AUC_0-24h_ and concentration at 24 hours postdose (*C*_24h_) stratified by dose. Horizontal lines indicates medians. Boxes represent IQRs while whiskers are the 2.5th and 97.5th percentiles. Dots represent individual values: n = 7, 600 mg; n = 40, 1200 mg (n = 30, day 3; n = 10, day 28). AUC, area under the curve.

**Figure 3** depicts the simulated plasma and CSF concentration time profiles for the typical participant in our cohort following a once daily dose of linezolid (600 or 1200 mg). The model-derived individual values for the steady-state AUC_0–24h_ and concentrations at 24 hours post-dose are shown in **Figure 4** and summarized in **Table 3**.

**Table 3.** Linezolid Model–Derived AUC for 24 Hours and Concentrations at 24 Hours Postdose

	Plasma		Cerebrospinal Fluid	
	1200 mg (n = 40)	600 mg (n = 7)	1200 mg (n = 40)	600 mg (n = 7)
AUC_0–24h_, mg·h/L	278 (87.3–762)	93.7 (66.7–167)	81.6 (19.7–234)	24.0 (6.55–56.8)
C_24_, mg/L	1.69 (0.154–13.5)	0.406 (0.0614–1.67)	1.32 (0.327–6.48)	0.369 (0.0495–1.02)

Data are presented as median (Min–Max.). Within-group comparisons are by daily dose (1200 and 600 mg).

Abbreviation: AUC_0–24h_, area under the curve from time 0 to 24 hours.

## DISCUSSION

Linezolid is being evaluated in several clinical trials as part of enhanced antimicrobial therapy for TBM. This is based on limited clinical evidence from small observational studies in TBM [10, 11] and reports of successful use in gram-positive CNS infection. However, there is scarce information on linezolid exposure in the CSF, especially among patients with TBM, a presumed requirement for clinical efficacy in this condition. We characterized the PK of linezolid in plasma and CSF from a cohort of South African patients with HIV-associated TBM. The extent of linezolid penetration into the CSF was on average ∼30% of plasma exposure and correlated with CSF protein concentrations; specifically, CSF penetration was higher in participants with higher CSF protein, reaching a maximal value of ∼37%. Coadministration with high-dose rifampicin (35 mg/kg/d), when comparing the duration of rifampicin treatment on day 3 vs day 28, did not have a significant effect on the PK of linezolid.

Several prior studies may help to contextualize our findings. A recent observational study reported CSF linezolid concentrations from 17 patients with TBM (only 1 with HIV) who received 600 mg of linezolid daily [25]. At 2 and 6 hours postdose, the median CSF concentrations were 0.90 and 3.14 mg/L and the CSF/serum ratios were 0.25 and 0.59, respectively. CSF linezolid concentrations were also reported from 2 small neurosurgical cohorts receiving 600 mg of linezolid intravenously every 12 hours. In the smaller study (n = 7), the mean observed CSF/plasma AUC ratio was 0.565 (n = 7); the mean (SD) AUC_0-∞_ after the first dose was 37.7 (23.9) mg·h/L; and AUC_0-12h_ after the fifth dose was 53.7 (50.3) mg·h/L. In the slightly larger study (n = 14), the mean observed CSF/plasma AUC ratio was 0.66, and the mean (SD) AUC in CSF was 101 (59.6) mg·h/L [26, 27]. Direct comparison is limited because of differences in population (HIV status, disease type and severity), dosing and administration, and drug assays. CSF/plasma concentration and AUC ratios should be cautiously interpreted in these prior studies [25–27] since observed CSF and plasma concentrations were compared at the same time points, not accounting for delay in distribution between the plasma and CSF. Despite having access to only a single CSF sample per visit (due to the invasive nature of lumbar puncture), using a model-based approach allowed us to describe the time course for linezolid entry into CSF. The limitation of sparse CSF sampling in our study was further mitigated by randomizing participants to different sampling times so that CSF samples could be obtained over the full dosing interval. Another limitation is the relatively high proportion of BLQ CSF samples, which gives high variability in the observed CSF concentrations that is reflected in the proportional error estimate for the CSF observations. To test the effect of these samples on our analysis, we conducted a sensitivity analysis after excluding the BLQ samples. Their exclusion mainly affected the estimate of the proportional error, which decreased but did not affect the estimates of the PPC and the equilibration half-life. Additionally, we performed a parametric bootstrap (stochastic simulation and estimation), which yielded uncertainty values in line with the values obtained from sampling/importance resampling, thus corroborating our confidence in the results [28].

Other studies have reported a relationship between the levels of CSF total protein (or albumin) and antituberculosis drugs in TBM [29, 30]. In a pediatric population, there was a linear relationship between log-transformed CSF protein concentration and the CSF penetration of rifampicin, with a 63% increase in the penetration coefficient for every 10-fold change in protein levels [29]. In a pediatric cohort with TBM, an exponential function was used to describe the relationship between CSF protein concentrations and the partition coefficient of rifampicin, where an increase of 1 g/L in CSF protein concentration resulted in a 1.28-fold increase in the partition coefficient [30].

There are 2 plausible, potentially overlapping, explanations for our finding of a correlation between CSF protein levels and extent of CSF linezolid partitioning. In a healthy state, the blood-CSF barrier is intact, and just a small fraction of plasma proteins can enter into the CNS, leaving only unbound drug fraction available for penetration into this compartment [7]. Inflammation associated with TBM may increase blood-CSF barrier permeability, causing plasma protein and total drug concentrations to be higher in the CSF. Another possible explanation for this relationship is higher endogenous CSF protein production from local inflammation, leading to alterations in CSF drug binding kinetics and higher concentrations of total drug in TBM. Quantification of free drug CSF concentrations may help to further delineate CSF protein–drug relationships.

Linezolid is provided with high-dose rifampicin (35 mg/kg/d) in ongoing efficacy trials for TBM. Because of prior reports of a drug-drug interaction between rifampicin and linezolid—plus the likelihood of a rifampicin dose effect on metabolizing enzyme activity [31], which could affect the linezolid plasma exposure and hence the CSF exposure—we investigated a potential effect of rifampicin on linezolid PK. In our study, there was no control group that received only linezolid without rifampicin to clearly identify a drug-drug interaction. However, estimated linezolid clearance in our cohort was comparable to that reported from patients receiving linezolid for drug-resistant pulmonary TB without concomitant rifampicin. In addition, since the maximal CYP induction effect of rifampicin occurs after at least a week [32], we investigated the effect of the duration of rifampicin therapy on linezolid PK (rather than rifampicin coadministration as a categorical covariate) and could not detect any significant trends. Furthermore, we found no relationship between 4β-OHC/cholesterol ratio or 4β-OHC alone (as a predictive biomarker of enzyme induction by rifampicin) and linezolid clearance or bioavailability. Our data indicate that even if rifampicin had an effect on linezolid exposures, it is unlikely to be clinically relevant.

In contrast to our findings, smaller studies among healthy volunteers and patients without TB have demonstrated a reduction in linezolid exposure when administered with rifampicin [14, 33–35]. This interaction has been variously attributed to either a large increase in the expression of the CYP3A4 isoenzyme, which typically has a small contribution to linezolid clearance [14], or the increased upregulation of linezolid intestinal secretion by rifampicin induction of P-glycoprotein [35]. There is no definitive evidence that linezolid is a substrate of P-glycoprotein; plus, it is mainly metabolized (∼68%) in the liver via morpholine ring oxidation, which is independent of the cytochrome system, with the remainder excreted unchanged via the kidneys [14].

As reported for patients with pulmonary TB, saturable elimination was observed at higher linezolid plasma concentrations, resulting in nonlinear PK [36]. Despite subtle differences in Michaelis-Menten elimination kinetics (*k*_m_), our estimates for CLmax and V are in line with previously published linezolid models [36–41]. Prior models based on patient data from non-TB [42] and pulmonary TB [43] samples included an empirical inhibition compartment to describe concentration- and time-dependent autoinhibition of elimination. We also tested this approach, but it did not result in a better model fit for our data; as such, clearance values estimated by these models are similar to ours. An overview of these models and a comparison with the current work are summarized in Supplementary Table 1.

Our analysis had several limitations. First, the sparse plasma sampling (3 samples) performed during the second PK visit does not allow for robust estimation of the nonlinearity in clearance, especially since only 7 participants were taking the reduced dose (600 mg). However, the model fit improved significantly (*P* < .001) when it included saturation of clearance with higher concentrations, supporting this conclusion. Second, a limitation of the PPC–CSF protein relationship in our model is that the minimum PPC was fixed to 0—meaning no linezolid enters the CSF—to prevent the estimation of negative PPC values, which are physiologically implausible. Yet, a CSF protein value of 0 is not observed in people where it varies between 0.2% and 0.5% of the total protein concentration of blood [44]. It is considered that 80% of CSF proteins originate in blood and that CSF proteins are diluted in a molecule size–dependent concentration gradient [45]. Finally, we did not undertake simulations to estimate probability of target attainment because a PK efficacy target is not established for TBM. While our simulations do suggest that 1200 mg of daily dosing will achieve linezolid concentrations above the critical concentration/minimum inhibitory concentration for *Mycobacterium tuberculosis*, it is important to note that this putative efficacy target is established in vitro under conditions that are completely different from CSF. Additionally, drug protein binding in the CSF is unknown, as is the relative free fraction of the active drug.

In conclusion, we successfully developed a population PK model for linezolid among adults with HIV-associated TBM, demonstrating that linezolid penetrates into the CSF, a surrogate compartment for site of disease in TBM, at potentially therapeutic concentrations, even with concomitant use of high-dose rifampicin. More investigations on the CSF protein–binding dynamics of linezolid are required to better understand its CSF partition. These findings support continued clinical evaluation of linezolid with rifamycins for the treatment of TBM in adults. Our model provides a platform that can be used for exploring alternative linezolid dosing strategies in TBM once effective and safe treatment targets are established for this condition.

## Supplementary Data

Supplementary materials are available at *The Journal of Infectious Diseases* online. Consisting of data provided by the authors to benefit the reader, the posted materials are not copyedited and are the sole responsibility of the authors, so questions or comments should be addressed to the corresponding author.

## Supplementary Material

jiad413_Supplementary_Data

## Notes

***Acknowledgments***. Computations were performed in facilities provided by the University of Cape Town's ICTS High Performance Computing team: https://ucthpc.uct.ac.za/. Study data were collected and managed in REDCap tools (research electronic data capture) hosted at the University of Cape Town. REDCap is a secure web-based software platform designed to support data capture for research studies [46, 47].

***Disclaimer.*** The content is solely the responsibility of the authors and does not necessarily represent the official views of the sponsors. The funders had no role in study design, data collection and analysis, decision to publish, or preparation of the manuscript. For the purpose of Open Access, the authors have applied a CC-BY public copyright license to any author accepted manuscript version arising from this submission.

***Financial support*.** This work was supported by the National Institutes of Health (K43TW011421 and U01AI170426 to S. W.); the Fogarty International Center (D43 TW010559 to M. T. A. for training in research); Wellcome Trust through core funding from the Wellcome Centre for Infectious Diseases Research in Africa (203135/Z/16/Z); a UCL Wellcome Trust PhD Programme for Clinicians Fellowship (175479 to A. D.); the Francis Crick Institute, which is funded by Wellcome Trust (CC2112), Cancer Research UK (CC2112), and UK Research and Innovation (CC2112; all to R. J. W.); the National Institutes of Health (R01145436 to R. J. W.); and Meningitis Now (R. J. W.). The University of Cape Town Clinical PK Laboratory is supported in part via the Adult Clinical Trial Group by the National Institute of Allergy and Infectious Diseases of the National Institutes of Health (UM1 AI068634, UM1 AI068636, and UM1 AI106701); and the International Maternal Pediatric Adolescent AIDS Clinical Trials Network (IMPAACT), with funding provided by the National Institute of Allergy and Infectious Diseases (U01 AI068632), the Eunice Kennedy Shriver National Institute of Child Health and Human Development, and the National Institute of Mental Health (AI068632). This research was funded in part by the Wellcome Trust. For the purpose of open access,we have applied a CC BY public copyright license to any Author Accepted Manuscript version arising from this submission.
