# Supplementary material for: Linezolid Population Pharmacokinetic Model in Plasma and Cerebrospinal Fluid Among Patients With Tuberculosis Meningitis
Source: J Infect Dis. 2023 Sep 22;229(4):1200–8. doi: 10.1093/infdis/jiad413 (PMC11011161; doi:10.1093/infdis/jiad413)
Supplement: jiad413_Supplementary_Data [file jiad413_supplementary_data.zip › Supplementary.material_V2_Clean.docx]

# Supplementary File

# Laboratory assays

## Linezolid

The CSF samples were processed with a protein precipitation extraction method using linezolid-d3 as the internal standard, followed by high-performance liquid chromatography with tandem mass spectrometry detection on a SCIEX API 3200 instrument. The analyte and internal standard were monitored at mass transitions of the protonated precursor ions 338.2 and 341.1 to the product ions 296.3 and 297.3 for linezolid and linezolid-d3, respectively. The calibration curve fitted a quadratic regression (weighted by 1/x) over the range of 0.1 to 20 mg/L. The accuracy of the quality control samples during sample analysis was between 103.5 and 105.5%, with precision of less than 2.1%.

**Free linezolid**

The free linezolid assay consisted of ultracentrifugation and dilution, followed by on-line solid phase extraction (SPE) and high-performance liquid chromatography with tandem mass spectrometry detection (LC-MS/MS). On-line SPE was achieved using a Restek Viva BiPh 5 µm, 50 mm x 1.0 mm column, and LC separation was achieved using an Agilent Poroshell 120 EC- C18 2.7 µm, 50 mm x 2.1 mm column, with a total run time of 6.5 minutes. A Sciex API 3200 mass spectrometer at unit resolution in the multiple reaction monitoring mode was used to monitor the transitions of the protonated precursor ions at 338.2 and 341.2 to the product ions at 296.2 and 297.2 for linezolid and linezolid-d3, respectively. Electrospray ionisation was used for ion production. The calibration curve was fitted with a quadratic regression (weighted by 1/x) based on peak area ratios over the range of 0.100 – 30.0 µg/mL. The accuracy of the quality control samples was between 101.1 and 104.4%, with a coefficient of variation of less than 8%.

## 4-beta hydroxy cholesterol (4β-OHC)

4β-OHC was measured with a high-performance liquid chromatography with tandem mass spectrometry assay in the Division of Clinical Pharmacology at the University of Cape Town. The extraction process involved a liquid-liquid extraction, which uses alkaline hydrolysis using potassium hydroxide and chemical derivatization using picolinic acid. Stable isotope labelled 4β-hydroxy cholesterol-d7 (4β-OHC-d7) was used to prepare calibration standards and quality control samples in human plasma. Endogenous 4β-OHC was measured using the surrogate analyte, 4β-OHC-d7. 4β-OHC -d4 was used as the internal standard. Chromatographic separation was done with gradient elution on a Gemini C6 Phenyl analytical column. A Sciex 5500 mass spectrometer at unit resolution in the multiple reaction monitoring acquisition mode was used to monitor the transition of protonated ions to their respective product ions. Electrospray ionization in the positive mode was used for ion production. The calibration curve fitted a quadratic regression (weighted by 1/x²) over the range of 2.00 to 500 ng/mL.

# Pharmacokinetic modelling

Nonlinear mixed-effects modelling in NONMEM^®^ 7.5 with first-order conditional estimation with eta-epsilon interaction (FOCE-I) was used to develop a population pharmacokinetic model that describes linezolid pharmacokinetics (PK) in both plasma and lumbar cerebrospinal fluid (CSF). Pirana 3.0.0 software was used for model management; Perl-speaks-NONMEM^®^ (PsN) 4.9.0 and R 4.0.4 via RStudio were used for post-processing NONMEM^®^ results and generating figures [1]. For the plasma model, the nonlinearity in clearance observed at higher doses was accounted for by a concentration-dependent $CL$ described by the following equation:

$$Vmax= CLmax\cdot Km$$

where $Vmax$ is the maximal elimination rate in mg/h, $CLmax$ is the maximal clearance in L/h apparent with linezolid plasma concentration ($Cp$) approximating 0, while $Km$ is the $Cp$ in mg/L at which the elimination is half of $Vmax$. Lag time and transit compartments were tested to capture the delay in the absorption process. Allometric scaling of clearance and volume parameters was tested as per Anderson and Holford [2] using the fixed power exponents of 0.75 for clearance and 1 for volume and either total body weight or fat-free mass (FFM) (calculated based on the formula in Janmahasatian et al.[3] ) as body size descriptors.

Between-subject, between-visit, and between-occasion variabilities were tested for the different plasma and CSF parameters. Each PK sampling day (day 3 and day 28) was considered as a separate visit. Each dose and its following samples were considered a separate occasion, therefore, the dose before the sampling visit along with the predose concentration were treated as a separate occasion from the dose administered during the PK visit and the following concentrations. Residual unexplained variability was described using a combined proportional and additive error model, with the additive error for all samples set to be at least 20% of the LLOQ. Concentrations below the lower limit of quantification (BLQ) were censored according to Beal’s M6 method, in which the last censored value in a series during the absorption phase and the first censored value in a series in the terminal phase was replaced with LLOQ/2 and the other censored values in a series were discarded [4]. To account for the larger level of uncertainty in the imputed censored values, their additive error was inflated by LLOQ/2. Also, the M3 method was tested as there was a large fraction of BLQ values in the CSF but it did not result in a meaningful difference in the parameter estimates. On the other hand, it caused longer run times and, most importantly, less stable final parameter estimates. For this reason, we proceeded with the M6 method [4].

The process of model development and covariate inclusion was guided by physiological plausibility, model fit diagnostics, and the drop in the objective function value (OFV). The likelihood ratio test for the drop in OFV was used to compare between nested models, assumed to be approximately χ^2^ distributed with n degrees of freedom, where n is the number of additional estimated parameters. A *p*-value of 0.05 was generally used for inclusion and 0.01 for retention. Model performance was evaluated by means of visual predictive checks (VPC). The VPC for the final model stratified into plasma and CSF concentrations is shown in **Figure S1**. Final parameters precision (95% confidence intervals) was obtained by sampling importance resampling (SIR) [5].

## Imputation of missing covariates

Missing covariates such as CSF protein, CSF albumin, and CSF glucose levels were imputed by the median. A different approach was used for the missing heights (necessary for fat-free mass calculation) since it was missing in 60% of the participants. Missing heights were imputed using multiple linear regression as suggested by Johansson and Karlsson [6]. In the first step, participant characteristics, namely sex, weight, and height from a study in a similar population [7] were used to develop a multiple linear regression model for height versus weight by sex and accounting for residual variability in heights. Secondly, this multiple linear regression model was used to estimate the missing heights in NONMEM using a random effect model as shown in the equation below:

$$Ht_{i}=\beta+\alpha.Wt_{i}.e^{\eta_{i}}$$

Where $Ht_{i}$ is the individual height in meters and $Wt_{i}$ is the individual weight in kilograms. $\beta$ and $\alpha$are the model mean intercept and slope respectively. $\eta_{i}$ is the random effect accounting for the individual difference from the mean values. The $\eta_{i}$ values are assumed to be normally distributed with mean zero and variance $\omega^{2}$.The values of $\beta$ and $\alpha$ are 1.51 and 0.00133 for females and 1.53 and 0.00281 for males respectively. the values of $\omega_{2}$ were 0.00215 and 0.00170 for females and male respectively. NONMEM implementation can be found in the NONMEM code provided.

## Effect compartment modelling for CSF concentrations

The CSF concentrations were modelled as dependent on plasma concentrations using an effect compartment, as previously proposed and implemented by Sheiner et al. [8] and Savic et al [9]. Effect compartments are assumed to have a negligible volume compared to the central compartment, with negligible drug transfer between the two compartments. The following differential equation summarizes the kinetics of the effect compartment:

$\frac{{dC}_{CSF}}{dt}=k_{plasma-CSF}\cdot\left( PPC\cdot C_{plasma}-C_{CSF} \right)$,

where $k_{plasma-Eff}$ is the first-order equilibration rate constant of the drug between the central compartment (i.e., plasma) and the effect compartment (i.e., CSF), $PPC$ is the pseudo-partition coefficient, $C_{plasma}$ and $C_{CSF}$ are the drug concentration at time $t$ in plasma or CSF, respectively. **Figure S2** shows the interpretability of the equilibration rate constant and the PPC in the context of effect compartment modelling approach.

**Table S1 Overview of Linezolid previously published popPK models and current work^a^.**

|  | Study population | Dose | CL (L/h) | V (L) | Q (L/h) | Vp (L) | Km  (mg/L) |
| --- | --- | --- | --- | --- | --- | --- | --- |
| Abdelwahab et al. 2021 [10] | DR-TB | 300 & 600 mg | 3.57 | 40.2 | - | - | - |
| Tietjen et al. 2021[11] | MDR-TB | 300, 350, 450, or 600 mg bid | 7.69 | 45.2 | - | - | - |
| Alghamdi et al. 2020 [12] | DS-TB & MDR-TB | 300 – 600 qd  600 mg qd or bid | 6.32 | 40.6 | - | - | - |
| Plock 2007 [13] | Healthy volunteers and septic patients | 600 mg bid | 11.1 | 20.2 | 75.0 | 28.9 | - |
| Meagher 2003^b^ [14] | Multidrug-resistant gram-positive infections | 600 mg bid | 6.85 | 39.3 | 9.09 | 23.6 | 1.46 |
| Imperial 2021^c^ [15] | XDR or TI/NR MDR-TB patients | linezolid dosages of 600 or 1200 mg total daily (twice- or once-daily) for 6 months | 7.9 | 49 | 0.8 | 14 | 16 |
| Current work^c^ | TBM patients with HIV | 1200 mg for 4 weeks followed by 600 mg | 7.25 | 40.8 | - | - | 27.2 |
| ^a^ None of the patients were co-administered with Rifampicin except for the current population.  ^b^ LZD popPK was described by parallel first-order and Michaelis-Menten elimination models. The CL value represents the average total clearance over the first week.  ^c^ LZD PK was described by a non-linear clearance (Michaelis-Menten).  XDR = extensively drug-resistant  Tl/NR MDR-TB =treatment-intolerant or nonresponsive TB patients | | | | | | | |

# Figures

**Figure S1:** Visual predictive check (VPC) (n=1000) showing plasma drug concentration versus time after dose for the final models stratified into plasma and cerebrospinal fluid. The dots are the original observations; the solid line is the median and the dashed lines are the 10^th^ and 90^th^ percentiles of the observed data; the shaded areas are the 95% confidence intervals of the same percentiles as simulated by the model. A suitably fitting model will have most of the observed percentiles within the simulated confidence intervals.

**Figure S2:** Demonstration on the interpretability of the equilibration rate constant and the PPC in the context of effect compartment modeling approach.

**Figure S3:** Free linezolid concentration (mg/L) versus the total linezolid concentration in the same sample. The slope of the regression equation represents the fraction of unbound linezolid The slope was estimated using generalized Deming regression with constant error assumption [16,17].

**Figure S4:** LOESS regression between linezolid fraction unbound and total linezolid concentrations(mg/L). There was no apparent trend between the two variables.

# NONMEM Control File

;; 1. Based on:

;Model desc: Final.model

;Settings for the memory of NONMEM

$SIZES PD=-1000 LVR=-150 LTH=-200 MAXFCN=10000000 LNP4=-150000

DIMTMP=1000

$PROBLEM LNZ_MODEL

$INPUT ID DV AMT …

$DATA dat.csv IGNORE=@

$ABBREVIATED DECLARE INTEGER NDOSE INTEGER MAX_ACCUM_DOSES

$SUBROUTINE ADVAN13 TRANS1 TOL=9 ATOL=9 ;SSTOL=6 SSATOL=6

$MODEL NCOMPS=5 ; NUMBER OF COMPARTMENTS

COMP=(ABS,DEFDOSE) ;1 GUT ABS

COMP=(CENTRAL) ;2 CENTRAL CMT

COMP=(CSF) ; 3 CSF CMT

COMP=(AUC_P) COMP=(AUC_CSF)

;initialization-of-theta(S)-from the previous run

$THETA (0,7.33136) ; 1 CLMAX (L/H)

$THETA (0,40.8985,100) ; 2 V (L)

$THETA (0,1.21424,3) ; 3 KA (1/H)

$THETA (0,0.211549,3) ; 4 MTT (H)

$THETA 1 FIX ; 5 BIO()

$THETA (0,5.67065,100) ; 6 NN ()

$THETA (0.01,0.21648,10) ; 7 PROP (%)

$THETA (1e-5,0.163042,2) ; 8 ADD (mg/L)

$THETA (0,26.2736) ; 9 KM

$THETA (0,0.197959,10) ; 10 KE0

$THETA (0,0.364727,1) ; 11 PPC

$THETA (0,0.3) ; 12 PROP_E []

$THETA 0 FIX ; 13 ADD_E [mg/L]

$THETA (0,1.18242,10) ; 14 BRK

$THETA 1 FIX ; 15 AMP

$THETA 0 FIX ; 16 INT

$OMEGA BLOCK(1)

0.00884734 ; 1 BSVCLMAX

$OMEGA BLOCK(1) FIX

0 ; 2 BSVV

$OMEGA BLOCK(1) FIX

0 ; 3 BSVKA

$OMEGA BLOCK(1) FIX

0 ; 4 BSVMTT

$OMEGA BLOCK(1) FIX

0 ; 5 BSVBIO

$OMEGA BLOCK(1)

0.772478 ; 6-10 BOVKA

$OMEGA BLOCK(1) SAME

$OMEGA BLOCK(1) SAME

$OMEGA BLOCK(1) SAME

$OMEGA BLOCK(1) SAME

$OMEGA BLOCK(1)

1.19782 ; 11-15 BOVMTT

$OMEGA BLOCK(1) SAME

$OMEGA BLOCK(1) SAME

$OMEGA BLOCK(1) SAME

$OMEGA BLOCK(1) SAME

$OMEGA BLOCK(1) FIX

0 ; 16-20 BOVBIO

$OMEGA BLOCK(1) SAME

$OMEGA BLOCK(1) SAME

$OMEGA BLOCK(1) SAME

$OMEGA BLOCK(1) SAME

$OMEGA 0.00215 FIX ; 21 Variance HTfemale

$OMEGA 0.00170 FIX ; 22 Variance HTmale

$OMEGA BLOCK(1)

0.0411842 ; 23-24 BVVCLMAX

$OMEGA BLOCK(1) SAME

$OMEGA BLOCK(1) FIX

0 ; 25 BSVKM

$OMEGA BLOCK(1) FIX

0 ; 26 BSVKE0

$OMEGA BLOCK(1) FIX

0 ; 27 BSVPPC

$OMEGA BLOCK(1) FIX

0 ; 28-29 BVVPPC

$OMEGA BLOCK(1) SAME

$SIGMA 1 FIX

;-------------------------------------------------------------------

$PK

;--------- Handling of missing data ----------

;- Imputation of HT and FFM all individuals -

IMP_HTM = ((0.00133*WTNEW) + 1.51)*EXP(ETA(21)) ;for females

IF (SEXF.EQ.0) IMP_HTM = ((0.00281*WTNEW) + 1.53)*EXP(ETA(22)) ;for males

IMP_FFMNEW = (37.99 * (IMP_HTM**2) * WTNEW) / (35.98 * (IMP_HTM**2) + WTNEW); for females

IF (SEXF.EQ.0) IMP_FFMNEW = (42.92 * (IMP_HTM**2) * WTNEW) / (30.93 * (IMP_HTM**2) + WTNEW);for males

;Adding Dose as covriate

IF(AMT.GT.0) DOSE_LZD = AMT

;-------Typical values of covariates

TVWT = 60 ;median wt from the data set

TVFFM = 45

;---------Allometric scaling and covariates

ALLMCL_WT = (WTNEW/TVWT)**0.75

ALLMV_WT = (WTNEW/TVWT)

IF(FFMNEW.NE.-99) ALLMCL_FFM = (FFMNEW/TVFFM)**0.75

IF(FFMNEW.EQ.-99) ALLMCL_FFM = (IMP_FFMNEW/TVFFM)**0.75

IF(FFMNEW.NE.-99) ALLMV_FFM = (FFMNEW/TVFFM)

IF(FFMNEW.EQ.-99) ALLMV_FFM = (IMP_FFMNEW/TVFFM)

;-------------------------------------------------------------------

; Covariate Testing

COV = CSF_PROTEIN

COV_MED = 0.995

IF (COV.LT.0) THEN

COV = COV_MED

ENDIF

BRK = THETA(14) ;lower limit must be 0

AMP = THETA(15)

INT = THETA(16) ;intercept - lower limit must be 0

SLP = (AMP - INT)/(BRK - 0)

SLP_AFTER = 0

IF(COV.LE.BRK) COV_EFF=(SLP*(COV-BRK))

IF(COV.GT.BRK) COV_EFF=(SLP_AFTER*(COV-BRK))

;PARAMTERS

TVCLMAX = THETA(1) *ALLMCL_FFM

TVV = THETA(2)*ALLMV_FFM ; Volume Typical Value WITH ALLOMETRIC SCALLING

TVKA = THETA(3) ; First Order oral abs Typical Value

TVMTT = THETA(4) ; Typical-VALUE-FOR-MTT

TVBIO = THETA(5) ; Typical BIO value

TVNN = THETA(6) ; NUMBER-OF-TRANSIT-COMPS

TVKM = THETA(9)

;EFFECT Parameters

TVKE0 = THETA(10)

TVPPC = THETA(11)*(1+COV_EFF)

;-------------------------------------------------------------------

;Defining ETA's

;BETWEEN SUBJETS VARIABILITY----------------------------------------

BSVCLMAX = ETA(1)

BSVV = ETA(2)

BSVKA = ETA(3)

BSVMTT = ETA(4)

BSVBIO = ETA(5)

;Defining Between Visit variability

BVVCLMAX = 0

IF (PK_VISIT == 3) BVVCLMAX = ETA(23) ;eta for visit day3

IF (PK_VISIT == 28) BVVCLMAX = ETA(24) ;eta for visit day28

;Defining Between OCC VARIABILITY-----------------------------------

BOVCL = 0

BOVKA = 0

BOVMTT = 0

BOVBIO = 0

BOVLAG = 0

;Defining Between OCC variability;

;OCCASION 1

IF (OCC==1) THEN

BOVKA = ETA(6)

BOVMTT = ETA(11)

BOVBIO = ETA(16)

;BVVCLMAX = ETA(23) ;eta for visit day3

BVVPPC = ETA(28) ;eta for visit day3

ENDIF

;OCCASION 2

IF (OCC==2) THEN

BOVKA = ETA(7)

BOVMTT = ETA(12)

BOVBIO = ETA(17)

;BVVCLMAX = ETA(23) ;eta for visit day3

BVVPPC = ETA(28) ;eta for visit day3

ENDIF

;OCCASION 3

IF (OCC==3) THEN

BOVKA = ETA(8)

BOVMTT = ETA(13)

BOVBIO = ETA(18)

;BVVCLMAX = ETA(24) ;eta for visit day28

BVVPPC = ETA(29) ;eta for visit day28

ENDIF

;OCCASION 4

IF (OCC==4) THEN

BOVKA = ETA(9)

BOVMTT = ETA(14)

BOVBIO = ETA(19)

;BVVCLMAX = ETA(24) ;eta for visit day28

BVVPPC = ETA(29) ;eta for visit day28

ENDIF

;OCCASION 5

IF (OCC==5) THEN

BOVKA = ETA(10)

BOVMTT = ETA(15)

BOVBIO = ETA(20)

;BVVCLMAX = ETA(23) ;eta for visit day3 (24h dose)

BVVPPC = ETA(28) ;eta for visit day3 (24h dose)

ENDIF

BSVKM = ETA(25)

BSVKE0 = ETA(26)

BSVPPC = ETA(27)

;-------------------------------------------------------------------

V = (TVV)*EXP(BSVV)

KA = (TVKA)*EXP(BSVKA+BOVKA)

MTT = (TVMTT)*EXP(BSVMTT+BOVMTT)

BIO = (TVBIO)*EXP(BSVBIO+BOVBIO)

NN = TVNN;*EXP(BSVNN)

CLMAX = TVCLMAX*EXP(BSVCLMAX+BVVCLMAX)

KM = TVKM*EXP(BSVKM)

VMAX = CLMAX*KM

;Effect parameters

KE0 = TVKE0 * EXP(BSVKE0)

PPC = TVPPC * EXP(BSVPPC+BVVPPC)

K23 = (CLMAX/V)*1E-12 ;negligible mass transfer from central to effect (CSF) compartment

;-------------------------------------------------------------------

; Transit code

F1=0 ; needed for this implementation of the transit compartment absorption

KTR = (NN+1)/MTT ; The number of actual transit compartments is NN+1, so this number can never be 0

IF (NEWIND/=2.OR.EVID>=3) THEN ; new individual, or reset event

; The values read here will be stored in TDOS and PD in this very PK call.

TNXD=TIME ; Time of the dose

PNXD=AMT ; Amount. If it's zero, the DE is deactivated.

TIMEDOSE = TIME

AMOUNTDOSE = AMT

ENDIF

TDOS=TNXD ; This will either save here the temporary values if it's a new individual...

PD=PNXD ; ...or the values which were read one record ahead during the execution of the previous record.

IF(AMT>0) THEN ; This reads one record ahead and stores the data to be used when running the following record

; IF(AMT.GT.0.AND.ALAG1.EQ.0) THEN ; Use this INSTEAD if there is ALAG, as it will also checks if the ALAG is not 0. Note that you normally do not want to include both ALAG and transit, this is a very exceptional case

TNXD=TIME

PNXD=AMT

ENDIF

; To speed up the computation, I calculate here all the non-time-varying quantities used in $DES

PIZZA = LOG(BIO*PD*KTR + 1E-12) - GAMLN(NN+1) ; without +0.00001, it won't work with ETAs in bioavailability

A_0(1) = 1E-12 ;ABS

A_0(2) = 1E-12 ;CENT

A_0(3) = 1E-12 ;CSF

A_0(4) = 1E-12

A_0(5) = 1E-12

;------------------------------------------------------------------

$DES

C2 = A(2)/V

TEMPO = T-TDOS ; this is time after dose for the transit, it should always be >= 0

KTT = 0

TRANSIT = 0

IF(PD.GT.0.AND.TEMPO.GT.0) THEN ; This happens only id PD>0, so only if a dose has been detected

KTT = KTR*(TEMPO)

TRANSIT = EXP(PIZZA+NN*LOG(KTT)-KTT)

ENDIF

DADT(1) = TRANSIT -KA*A(1)

DADT(2) = KA*A(1) - ((VMAX/(KM+C2))/V)*A(2)

DADT(3) = KE0*(PPC*C2 - A(3)) ;A(3) IS ACTUALLY CONC IN EFFECT CMT

DADT(4) = C2;*TSS1

DADT(5) = A(3);*TSS1

$ERROR

; In the dataset, flag CENSORED values:

; CENSORED==0 means that the value given by lab isn't censored

; CENSORED==1 means that the DV value was CENSORED and we would like to use it in the model fit

; CENSORED==2 same as above but we don't want it to affect the model fit but we leave it there for diagnostics

LLOQ_LZD=0.100

LLOQ_P = 0.100

LLOQ_E = 0.100

CENS_THR = LLOQ_LZD

CP = A(2)/V

CE = A(3)

IPRED_P = CP

PROP_P = IPRED_P*THETA(7)

ADD_P = THETA(8) + (0.2*CENS_THR)

IF(ICALL.NE.4.AND.BLQ_LNZ==1.AND.DVID==1) THEN

ADD_P = ADD_P + (LLOQ_P/2)

ENDIF

W_P = SQRT((ADD_P)**2 + (PROP_P)**2)

IPRED_E = CE

PROP_E = IPRED_E*THETA(12)

ADD_E = THETA(13) + (0.2*CENS_THR)

IF(ICALL.NE.4.AND.BLQ_LNZ==1.AND.DVID==2) THEN

ADD_E = ADD_E + (LLOQ_E/2)

ENDIF

W_E = SQRT((ADD_E)**2 + (PROP_E)**2)

ERROR_P = W_P * ERR(1)

ERROR_E = W_E * ERR(1)

;Redefine IPRED & weighting

IPRED = IPRED_P

W = W_P

ERROR_TERM = ERROR_P

IF (DVID==2) THEN

IPRED = IPRED_E

W = W_E

ERROR_TERM = ERROR_E

ENDIF

; Protective code

IF (W.LE.0.000001) W=0.000001

IRES = DV-IPRED

IWRES = IRES/W

Y = IPRED + ERROR_TERM

; To prevent simulation (ICALL==4) of negative values, set a positive lower bound for Y, so that VPCs in the log-scale can be plotted

IF (DVID==1.AND.ICALL==4.AND.Y<=LLOQ_P) Y=LLOQ_P/2

IF (DVID==2.AND.ICALL==4.AND.Y<=LLOQ_E) Y=LLOQ_E/2

;-------------------------------------------------------------------

IF(AMT>0) THEN

TIMEDOSE = TIME

AMOUNTDOSE = AMT

ENDIF

TAD2 = TIME-TIMEDOSE

TSOD = TAD2

IF(OCC.EQ.1.OR.OCC.EQ.3) TSOD = 24 - TAD2

;RETRIEVE AMOUNT IN EACH COMPARTMENT-------------------------------

A_GUT = A(1)

A_CENT = A(2)

A_CSF = A(3)

AUC_P = A(4)

AUC_CSF = A(5)

VARCLMAX = BSVCLMAX + BVVCLMAX

VARPPC = BSVPPC + BVVPPC

CONC_MOD = A(2)/V

;-------------------------------------------------------------------

$ESTIMATION METHOD=1 INTER MAXEVAL=9999 PRINT=1 NOABORT NSIG=3 SIGL=6

NONINFETA=1 ETASTYPE=1

$TABLE …

# References

1. Keizer RJ, Karlsson MO, Hooker A. Modeling and simulation workbench for NONMEM: Tutorial on Pirana, PsN, and Xpose. CPT Pharmacometrics Syst Pharmacol. 2013/07/10. Department of Pharmaceutical Biosciences, Pharmacometrics research Group, Uppsala University, Uppsala, Sweden.; **2013**; 2(6):1–9.

2. Anderson BJ, Holford NHG. Mechanism-based concepts of size and maturity in pharmacokinetics. Annu Rev Pharmacol Toxicol. **2008**; 48:303–332.

3. Janmahasatian S, Duffull SB, Ash S, Ward LC, Byrne NM, Green B. Quantification of Lean Bodyweight. Clin Pharmacokinet. **2005**; 44:1051–1065.

4. Beal SL. Ways to fit a PK model with some data below the quantification limit. J Pharmacokinet Pharmacodyn. **2001**; 28(5):481–504.

5. Dosne AG, Bergstrand M, Harling K, Karlsson MO. Improving the estimation of parameter uncertainty distributions in nonlinear mixed effects models using sampling importance resampling. J Pharmacokinet Pharmacodyn. **2016**; 43(6):583–596.

6. Johansson ÅM, Karlsson MO. Multiple imputation of missing covariates in NONMEM and evaluation of the method’s sensitivity to η-shrinkage. AAPS J. **2013**; 15(4):1035–1042.

7. Brust JCM, Gandhi NR, Wasserman S, et al. Effectiveness and Cardiac Safety of Bedaquiline-Based Therapy for Drug-Resistant Tuberculosis: A Prospective Cohort Study. Clin Infect Dis An Off Publ Infect Dis Soc Am [Internet]. Oxford University Press; **2021** [cited 2023 Aug 28]; 73(11):2083. Available from: /pmc/articles/PMC8664482/

8. Sheiner LB, Stanski DR, Vozeh S, Miller RD, Ham JS, Francisco C. Simultaneous modeling of pharmacokinetics and pharmacodynamics: Application to d-tubocurarine. 1979.

9. Savic R, Ruslami R, Hibma J, et al. Pediatric tuberculous meningitis: Model-based approach to determining optimal doses of the anti-tuberculosis drugs rifampin and levofloxacin for children. Clin Pharmacol Ther [Internet]. **2015**; 98(6):622–629. Available from: https://onlinelibrary.wiley.com/doi/10.1002/cpt.202

10. Tuberculosis D, Abdelwahab MT, Wasserman S, Brust JCM, Dheda K, Wiesner L. Linezolid Population Pharmacokinetics in South African Adults. American Society for Microbiology;

11. Tietjen AK, Kroemer N, Cattaneo D, Baldelli S, Wicha SG. Population pharmacokinetics and target attainment analysis of linezolid in multidrug‐resistant tuberculosis patients. Br J Clin Pharmacol. **2021**; (February):1–10.

12. Alghamdi WA, Al-Shaer MH, Peloquin CA. Protein binding of first-line antituberculosis drugs. Antimicrob Agents Chemother [Internet]. American Society for Microbiology; **2018** [cited 2022 Nov 10]; 62(7). Available from: https://journals.asm.org/journal/aac

13. Plock N, Buerger C, Joukhadar C, Kljucar S, Kloft C. Does linezolid inhibit its own metabolism? - Population pharmacokinetics as a tool to explain the observed nonlinearity in both healthy volunteers and septic patients. Drug Metab Dispos. **2007**; 35(10):1816–1823.

14. Meagher AK, Forrest A, Rayner CR, Birmingham MC, Schentag JJ. Population pharmacokinetics of linezolid in patients treated in a compassionate-use program. Antimicrob Agents Chemother. **2003**; 47(2):548–553.

15. Imperial MZ, Nedelman JR, Conradie F, Savic RM. Proposed Linezolid Dosing Strategies to Minimize Adverse Events for Treatment of Extensively Drug-Resistant Tuberculosis. Clin Infect Dis [Internet]. **2022**; 74(10):1736–1747. Available from: https://academic.oup.com/cid/article/74/10/1736/6380680

16. Deming WE. Statistical adjustment of data. Stat. Adjust. data. Oxford, England: Wiley; 1943.

17. Linnet K. Performance of Deming regression analysis in case of misspecified analytical error ratio in method comparison studies. Clin Chem [Internet]. **1998**; 44(5):1024–31. Available from: http://www.ncbi.nlm.nih.gov/pubmed/9590376
