# Supplementary figures and images for: Linezolid Population Pharmacokinetic Model in Plasma and Cerebrospinal Fluid Among Patients With Tuberculosis Meningitis
Source: J Infect Dis. 2023 Sep 22;229(4):1200–8. doi: 10.1093/infdis/jiad413 (PMC11011161; doi:10.1093/infdis/jiad413)

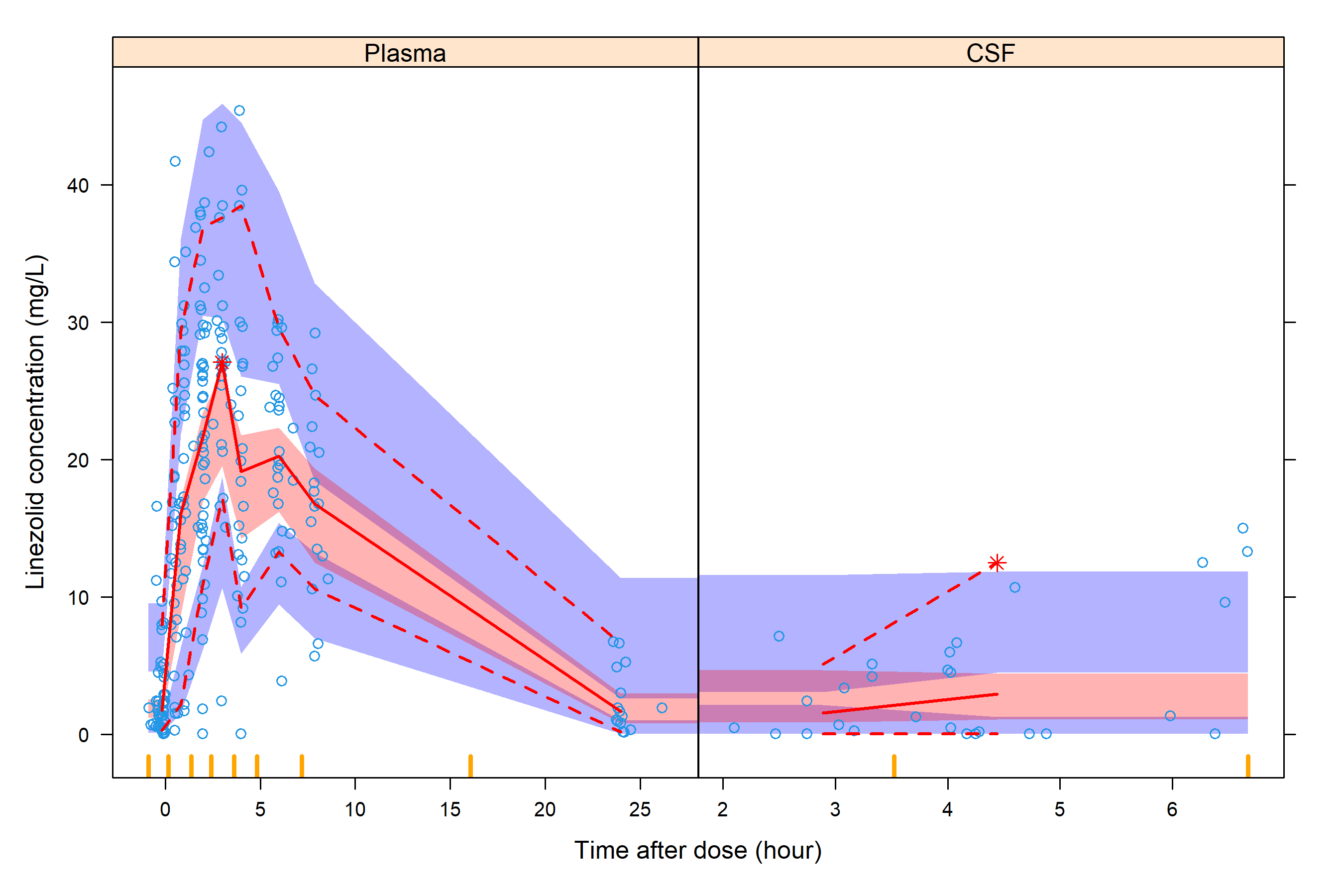

Supplement: jiad413_Supplementary_Data [file jiad413_supplementary_data.zip › FigS1VPC.png]

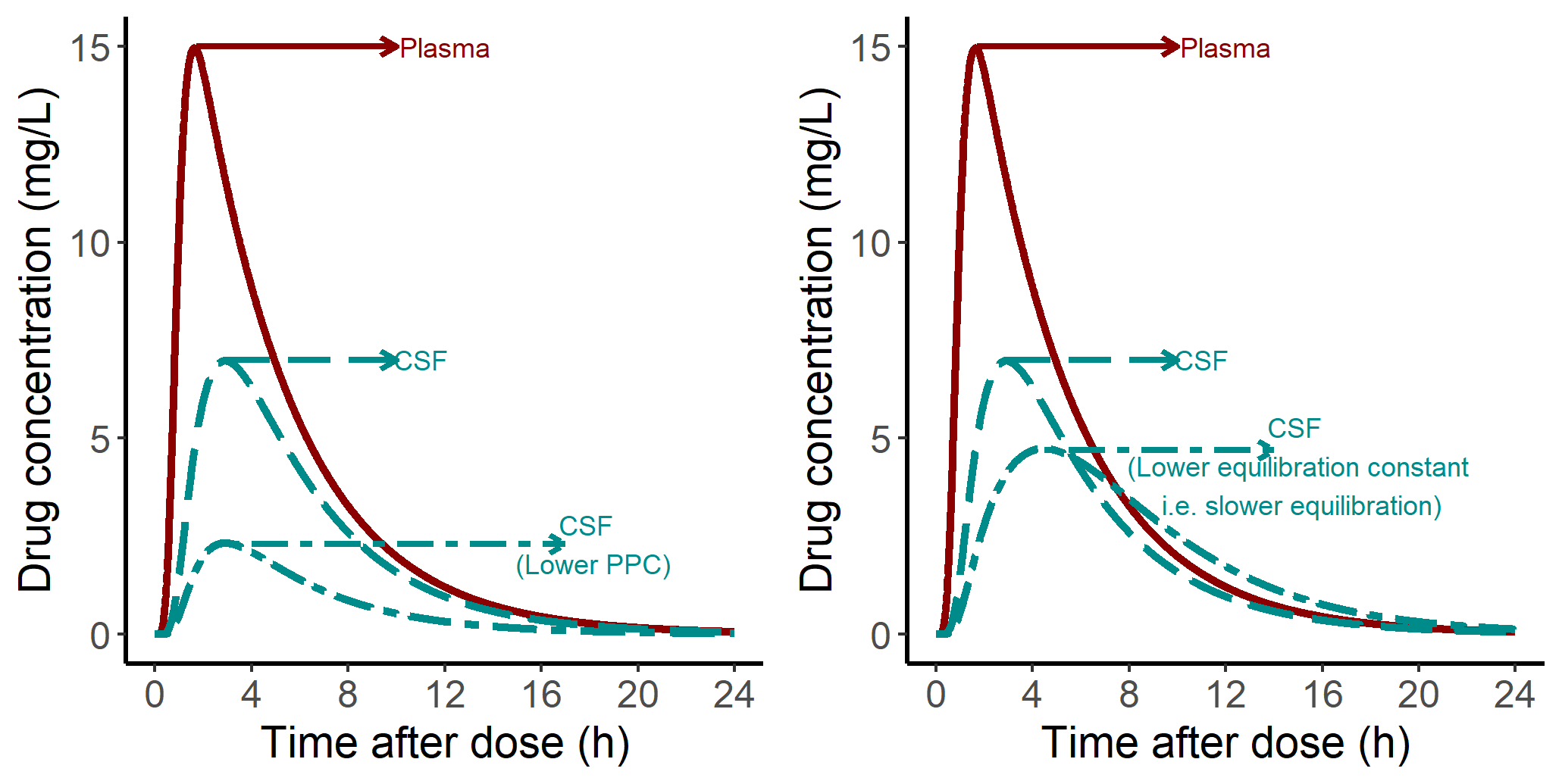

Supplement: jiad413_Supplementary_Data [file jiad413_supplementary_data.zip › FigS2_demo_fig.png]

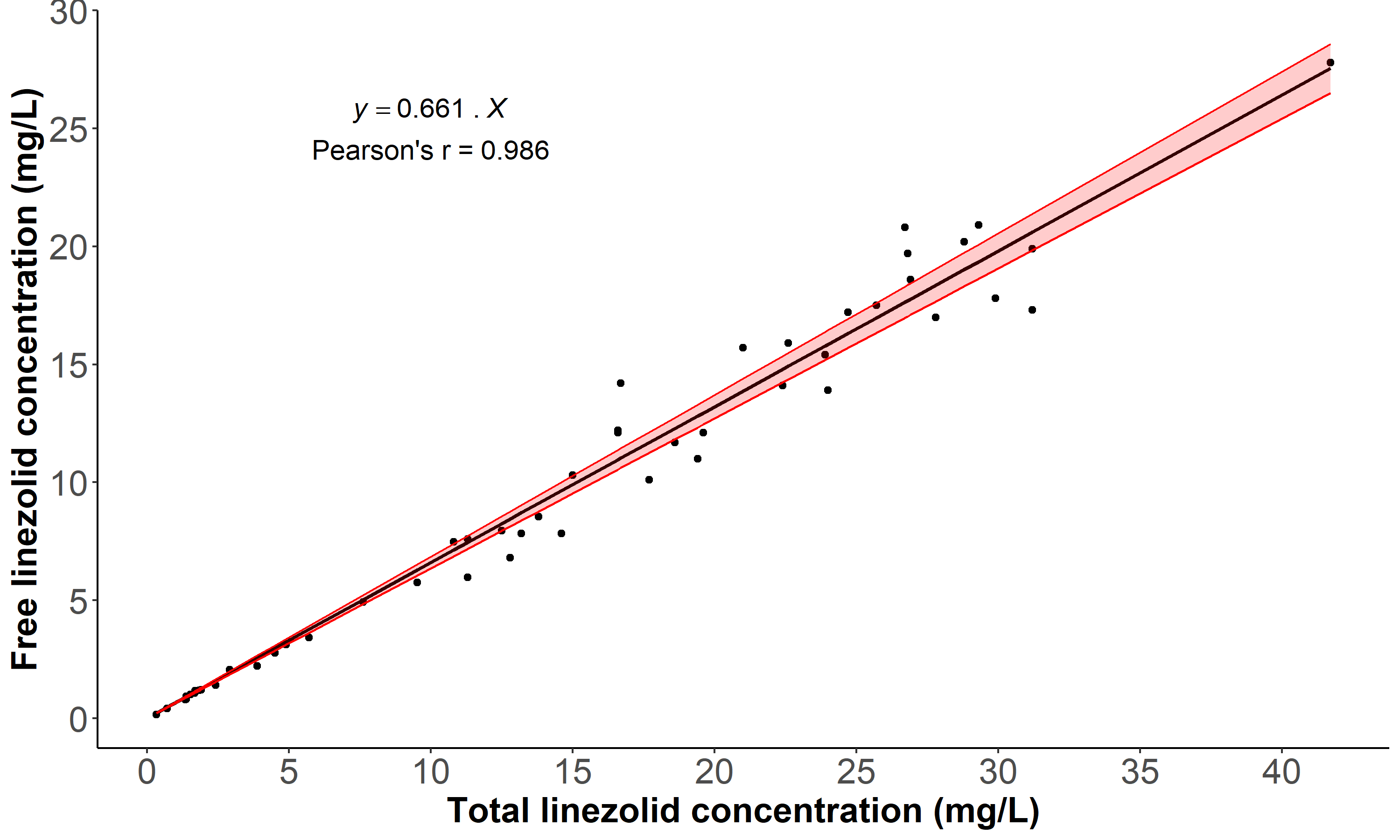

Supplement: jiad413_Supplementary_Data [file jiad413_supplementary_data.zip › FigS3.binding.png]

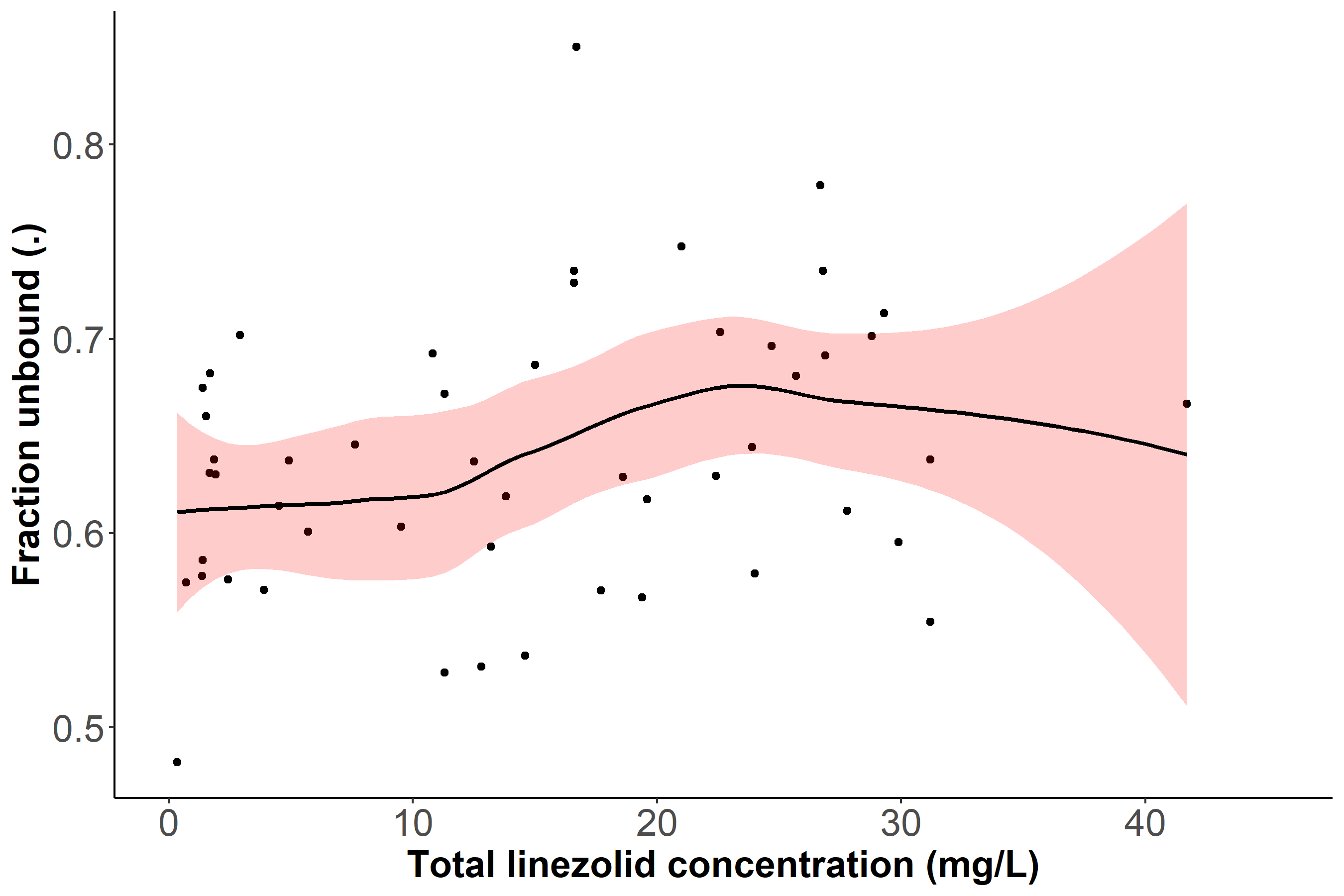

Supplement: jiad413_Supplementary_Data [file jiad413_supplementary_data.zip › FigS4.binding.png]
